# ESI-LC-MS based-metabolomics data of mangosteen (*Garcinia mangostana* Linn.) fruit pericarp, aril and seed at different ripening stages

**DOI:** 10.1016/j.dib.2018.02.033

**Published:** 2018-02-15

**Authors:** Siti Farah Mamat, Kamalrul Azlan Azizan, Syarul Nataqain Baharum, Normah Mohd Noor, Wan Mohd Aizat

**Affiliations:** Metabolomics Research Laboratory, Institute of Systems Biology (INBIOSIS), Universiti Kebangsaan Malaysia (UKM), 43600 Bangi, Selangor, Malaysia

**Keywords:** Ripening, *Garcinia mangostana* Linn., Metabolomics, ESI-LC-MS

## Abstract

Fruit ripening is a complex phenomenon involving a series of biochemical, physiological and organoleptic changes. Ripening process in mangosteen (*Garcinia mangostana* Linn.) is unique of which the fruit will only ripen properly if harvested during its middle stage (emergence of purple/pink colour) but not earlier (green stage). The knowledge on the molecular mechanism and regulation behind this phenomenon is still limited. Hence, electrospray ionization liquid chromatography mass spectrometry (ESI-LC-MS) based metabolomics analysis was applied to determine the metabolome of mangosteen ripening. Specifically, mangosteen pericarp, aril and seed were collected at four different ripening stages (stage 0: green, stage 2: yellowish with pink patches, stage 4: brownish red and stage 6: dark purple) and subjected to metabolite profiling analysis. The data provided in this article have been deposited to the EMBL-EBI MetaboLights database (DOI: 10.1093/nar/gks1004. PubMed PMID: 23109552) with the identifier MTBLS595. The complete dataset can be accessed here https://www.ebi.ac.uk/metabolights/MTBLS595.

**Specifications**

**Table:** 

Subject area	Biology
More specific subject area	Metabolomics
Type of data	Analysed ESI-LC-MS data
How data was acquired	Mass spectrometry data was acquired from MicrOTOF-Q III (Bruker Daltonic) using an ESI negative ionisation modes
Data format	Analysed data in.xlsx format
Experimental factors	Metabolites were extracted from the whole fruit of stage 0 and from the pericarp, aril and seed tissues of the three other ripening stages (stages 2, 4 and 6)
Experimental features	Plant materials were extracted using methanol acidified with formic acid solvent and 100 ppm (±)-naringenin (*m/z*: 272.06) was used as an internal standard. Sample was analysed with ESI-LC-MS system and processed using Profile Analysis software
Data source location	Universiti Kebangsaan Malaysia (UKM), Bangi, Selangor, Malaysia (2°55′09.0″N 101°47′04.8″E)
Data accessibility	Available with article (Supplementary Table 1)

**Value of the Data**

• The data consists of molecular ions (*m/z*) value and its corresponding intensities, in which were detected in mangosteen pericarp, aril and seed at four different ripening stages (stages 0, 2, 4 and 6)
• This enables determination of the spatial and temporal metabolite changes that occur during mangosteen fruit ripening
• Metabolite profiles of different parts of the fruit at different ripening stages are of key importance towards understanding the ripening process of mangosteen fruit
• The data can complement available mangosteen transcriptomic data for further analysis [1]

## 1 Data

Data was reported in.xlsx format comprising of the molecular ions (*m/z)* value with its corresponding retention time (RT) and intensities. All raw data were processed and bucketed using ProfileAnalysis (Bruker Daltonics, Germany). Specifically, Find Molecular Features (FMF) was applied to the raw data under these parameters: signal/noise (S/N) threshold was set to 5, correlation coefficient threshold was set to 0.7, minimum compound length was set to 10 and smoothing width was set to 3. The data was evaluated in a time range from 0.00 minutes to 25.02 minutes and in a mass ranges from m/z of 50 to 1000. Advanced bucketing was employed using time alignment as a parameter. Obtained datasets then were normalized against internal standard ((±)-naringenin, 100 ppm)), and subjected to log transformation (log 10) using MetaboAnalyst 3.0.

## 2 Experimental design, materials and methods

### 2.1 Chemicals and reagents

Analytical-grade methanol was purchased from Merck (Germany) and formic acid was purchased from APS Ajax Finechem (Auburn, Australia). (±)-Naringenin was purchased from Sigma-Aldrich, St. Louis, USA.

### 2.2 Plant materials preparation

Mangosteen fruits were harvested during its seasonal period from April until August 2016 according to the Malaysian Maturity Indices [2]. Four stages were selected as the representatives of mangosteen ripening stages as follow; stage 0: green, stage 2: yellowish with pink patches, stage 4: brownish red and stage 6: dark purple. Mangosteen fruits at stage 0 were sampled as whole fruits as the pericarp, aril and seed were inseparable at this stage [2]. For the other ripening stages (stages 2, 4 and 6) fruits were separated into pericarp, aril and seed. All fruit samples were then grounded into fine powder using laboratory-grade blender with the aid of liquid nitrogen. Samples were kept at -80°C until further analysis.

### 2.3 Metabolite extraction methods

Three biological and three technical replicates were prepared for each sample group (N=90). Metabolite extraction was performed based on [3] with slight modifications. Freshly prepared ice-cold extraction solution (75% methanol acidified with 0.1% formic acid, 599:1 v/v) in a volume per fresh weight ratio of 3:1 was added into each sample tube containing 200 mg of freeze-dried sample powder. Sample was immediately vortexed for 10 seconds and sonicated at maximum frequency (40 kHz) in ultra-sonication bath (20 minutes, 25 °C). Then, sample was centrifuged at maximum speed (16,100 × *g,* 10 minutes) at room temperature. Supernatant was collected and transferred into a new tube. Approximately 1 ml of sample including 100 ppm of (±)-naringenin as an internal standard was sent for the ESI-LS-MS analysis.

### 2.4 ESI-LC-MS parameter

Compound separation was performed on Thermo Scientific C18 column (Acclaim^™^ Polar Advantage II, 3 × 150 mm, 3 um particle size) on an Thermo Scientific Dionex UltiMate 3000 UHPLC system. The volume of sample for injection was 1.0 μl. Gradient elution was performed for 22 minutes (0.4 ml/min, 40 °C) using H_2_O + 0.1% formic acid (A) and 100% acetonitrile (B). The gradient started at 5% solvent B (0–3 minutes); 80% solvent B (3–10 minutes); 80% solvent B (10–15 minutes) and 5% solvent B (15–22 minutes) [4]. High resolution mass spectrometry was carried out using a MicroTOF QIII Bruker Daltonic system (Bruker, Germany). ESI negative ionization mode was performed with the following settings; capillary voltage: 4500 V, nebulizer pressure: 1.2 bar, drying gas: 8 l/min at 200 °C, mass range: 50–1000*m/z*.

### 2.5 Mass spectrometry data treatment

The MS raw data was obtained in*.d* format from Bruker Compass DataAnalysisViewer version 4.2 (Bruker Daltonics, Germany) before imported into ProfileAnalysis 2.0 software (Bruker Daltonic, Germany) for data bucketing. Compound detection was performed by subjecting the raw data to Find Molecular Features (FMF) using following parameter: S/N threshold: 5, correlation coefficient: 0.7, minimum compound length was: 10, smoothing width: 3. Compound bucket table was calculated using advanced bucketing feature using the parameters from time alignment. Time range was set to 0.00 minutes until 25.02 minutes and in a mass ranges from m/z of 50 to 1000. The comprehensive list of *m/z* values was extracted and submitted to the EMBL-EBI MetaboLights database (https://www.ebi.ac.uk/metabolights/), an open-source open-access repository for metabolomics studies [5]. The data was also uploaded to MetaboAnalyst 3.0 server (www.metaboanalyst.ca) for normalization according to the following settings; normalization feature: internal standard ((±)-naringenin, m/z: 271.06 m/z, RT: 4.87 minutes)), transformation: log transformation, scaling: pareto scaling [6]. Sample group with missing value was filtered by replacing with a small value (half of the minimum positive value of the data).

## Appendix A Supplementary material

**Supplementary Table 1** List of *m/z* value with their respective retention time (RT) and normalized intensities. The data was organized according to three biological replicates with three technical replicates for each sample groups (N=90). Mangosteen fruit from stage 0 (whole fruit) and mangosteen pericarp, aril and seed (stages 2, 4 and 6) were extracted using methanol acidified with formic acid and analysed using ESI-LC-MS system. S0: stage 0, S2: stage 2, S4: stage 4, S6: stage 6, B1: biological replicate 1, B2: biological replicate 2 and B3: biological replicate 3, T1: technical replicate 1, T2: technical replicate 2, T3: technical replicate 3.
